# A Companion to the United States Pharmacopœia

**Published:** 1884-05

**Authors:** 


**A Companion to the United States Pharmacopœia.** A practical commentary and key to the latest edition of the Pharmacopœia, and containing the descriptions, properties, uses and doses of all official and numerous unofficial drugs and preparations in current use in the United States. Designed as a ready reference book and working manual for pharmacists, physicians and students. By OSCAR OLDBERG, Ph. D., member of the Committee of Revision and Publication of the Pharmacopæia of the United States of America; author of the "Unofficial Pharmacopœia," "The Metric System in Medicine," etc.; formerly Medical Purveyor of the United States Marine Hospital Service, and Professor of Materia Medica in the National College of Pharmacy, Washington, D. C., etc., and OTTO A. WALL, M. D., Ph. G., Professor of Materia Medica, Therapeutics and Pharmacy in the Missouri Medical College, and of Materia Medica and Botany in the Saint Louis College of Pharmacy; member of the Committee of Revision and Publication of the Pharmacopœia of the United States of America, etc. Wm. Wood & Co., New York. 1884. J. H. Matteson, agent, No. 11 East Eagle street, Buffalo, N. Y. Price, muslin, $6.75; leather, $7.50.

This work gives evidence of unremitting care and labor on the part of its most competent authors, and is believed to be the most practically useful book of its class. It contains all the essential and necessary matter of the more cumbersome dispensatories without the immense mass of information they contain, which is rarely called for, and which makes it more difficult to readily find the practical points. Among the specially valuable features we rate the excellent articles on the newer remedies, *e. g.*, convallaria, quebracho, coto, etc., the admirable illustrations representing actual specimens, by which, at a glance, a better idea of many drugs can be had than by pages of verbal description. Its 1,200 pages contain more than 650 engravings. Its graphic descriptions enable the physician to distinguish between drugs of good, bad or indifferent quality, while to the pharmacist, the practical nature and the completeness of the work makes it invaluable.

